# Polyaniline Functionalized Graphene Nanoelectrodes for the Regeneration of PC12 Cells via Electrical Stimulation

**DOI:** 10.3390/ijms20082013

**Published:** 2019-04-24

**Authors:** Zheng Zheng, Libin Huang, Lu Yan, Feng Yuan, Lefeng Wang, Ke Wang, Tom Lawson, Mimi Lin, Yong Liu

**Affiliations:** 1 Laboratory of Nanoscale Biosensing and Bioimaging, School of Ophthalmology and Optometry, School of Biomedical Engineering, Wenzhou Medical University, 270 Xueyuanxi Road, Wenzhou 325027, China; zhengzheng1991@sohu.com (Z.Z.); libinhuang@sohu.com (L.H.); yanlu2017@wmu.edu.cn (L.Y.); fengyuanjy@sohu.com (F.Y.); lefengwang1994@gmail.com (L.W.); kewang72@foxmail.com (K.W.); 2 ARC Center of Excellence for Nanoscale BioPhotonics, Macquarie University, Sydney, NSW 2109, Australia; tomxlawson@gmail.com

**Keywords:** polyaniline, graphene, neuroregeneration, PC12 cells, electrical stimulation

## Abstract

The regeneration of neurons is an important goal of neuroscience and clinical medicine. The electrical stimulation of cells is a promising technique to meet this goal. However, its efficiency highly depends on the electrochemical properties of the stimulation electrodes used. This work reports on the preparation and use of a highly electroactive and biocompatible nanoelectrode made from a novel polyaniline functionalized graphene composite. This nanocomposite was prepared using a facile and efficient polymerization-enhanced ball-milling method. It was used to stimulate the growth of PC12 cells under various electrical fields. The enhanced growth of axons and improved wound regeneration of PC12 cells were observed after this treatment, suggesting a promising strategy for neuro traumatology.

## 1. Introduction

Neuro traumatology can impact the whole nervous system. It is mainly caused by neural traumata, which itself is caused by traffic accidents, tumor damages, and side effects from neurosurgery [1]. Neural damages always lead to the physical and psychological incapacitation of patients and reduce their ability to work [2]. Thus, the regeneration of damaged neurons is a fundamental goal of neuroscience and clinical medicine.

Electrical stimulation (ES) is considered a potentially useful therapeutic for the nervous system and for wound healing [3,4]. Electro-excitable tissues (such as peripheral nerves, the central nervous system, the deep brain, and muscles) are considered useful candidates for clinical treatment via ES. When their threshold is reached under an electrical field, portions of the axon membranes of electro-excitable tissues are artificially depolarized. This causes action potential across axons [5]. Although the structural and functional recovery of a damaged nervous system depends upon a variety of factors, reports elsewhere support ES use for the promotion of the neurite growth of treated nerve cells. They reported improved survival rates and better functional preservation of the central nervous system after injury [6].

A fundamental issue in the development of electrically stimulated regeneration of neurons is the correct selection of electrodes that have enough of a safe-charge-injection limit (Q_inj_, i.e., electrochemical capacitance). However, the regular clinical use of ES was thwarted because of the absence of suitable stimulation electrodes with a high Q_inj_, good biocompatibility, and long-term stability [7]. For example, ES of peripheral nerves requests a relatively high charge per pulse, i.e., high charge storage capacity across a relatively small sized electrode [8]. Conventional metal-based stimulation electrodes, such as platinum or metal oxide (i.e., iridium oxide) coated electrodes [9], were found to be not suitable for neural stimulation due to their poor electroactivity. To this end, electrically conducting polymers (CPs) were incorporated in reports elsewhere, as they had better electroactivity properties than conventional neural electrodes [10]. Recently, graphene based nanoelectrodes were described that provided promising electrical, mechanical, and biocompatible properties [11,12]. The two-dimensional (2D) π–π conjugated plane of the graphene matrix enables a fast electron-delivery channel between the electrode and the cells. This increases the cell response to an applied electrical field during cell regeneration. The large surface area of graphene helps to integrate it with cells. Unfortunately, graphene-only electrodes have a low electroactivity and so are not suitable for use in ES. Using nitrogen-doping of graphene was also observed to improve the electroactivity of graphene-based nanoelectrodes [13]. The incorporation of a range of nitrogen-containing polymers (including conducting polymers) with graphene was reported as an efficient way to enhance the electroactivity of graphene [14].

In this work, we demonstrate a facile but efficient way to prepare a conducting polymer (e.g., polyaniline), functionalized with graphene via a polymerization enhanced edge-functionalization ball-milling (EFBM) method. Along with other groups from reports elsewhere [15,16], we discovered that the EFBM method can provide an eco-friendly, low cost, facile, and efficient technique for preparing chemical functional groups (such as carboxyl- and carbonyl-) modified graphene for multifunctional applications. We also reported that nitrogen-containing polymers, such as chitosan, can be added to form highly electroactive nitrogen-doped graphene via the EFBM method [14]. In the present research, the EFBM technique was further improved using a monomer polymerization process. Typically, a nitrogen-containing monomer, such as aniline, was ball milled together with graphite powder. Using this process, the edge-functionalization of aniline with graphite layers (via active nitrogen sites) is formed first. Then, polymerization of aniline occurs because of the heat from the milling friction. The formation of polymer chains increases the layered distance of the graphite and further helps the exfoliation of graphene nanosheets during milling. The formed polyaniline functionalized graphene (PANI-G) has various useful properties. These include high electroactivity (because of its edge nitrogen-doped graphene and conducting polymers), excellent mechanical and electrical properties (because of its graphene matrix), and a high biocompatibility. These properties make the PANI-G a potentially useful nanoelectrode for ES.

The as-synthesized PANI-G was then applied here to the growth of PC12 cells. PC12 is a cell line derived from the pheochromocytoma of the rat adrenal medulla. PC12 cells are frequently used as a cell model for neuronal studies since PC12 cells can easily differentiate into neuron-like cells [17]. In this work, an alternating electrical field was applied to the PC12 cell-cultured PANI-G nanoelectrode. This significantly increased the axon length, by 60%, with no adverse impact on the cell density and enhanced the wound regeneration ability of PC12 cells. This may show that the as-synthesized nanoscale ES system could be useful for the regeneration of neurons.

## 2. Results

### 2.1. Characterization of PANI-G

**Figure 1:**
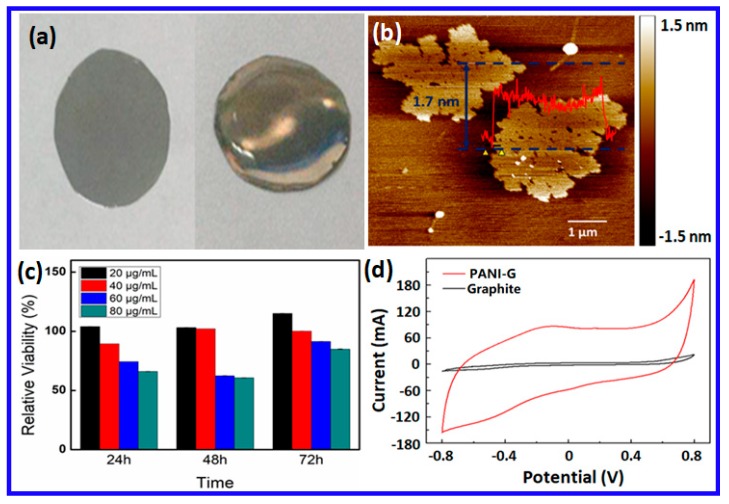
(**a**) Digital photo of the flexible ITO film (left), compared to the PANI-G coated ITO film; (**b**) AFM micrograph of the as-prepared PANI-G.; (**c**) CCK-8 results of the PA PANI-G treated PC-12 cells; (**d**) Cyclic voltammograms of the PANI-G coated on the glassy carbon electrode, compared to the pristine graphite treated by ball milling under identical conditions. The error bars in (**c**) are too small to be visible since the difference between various groups was very small. The presence of error bars can be seen in Figure S5 which magnified the corresponding parts of (**c**).

As described, PANI-G was prepared by ball-milling the mixture of aniline and graphite. The nanomaterials produced were dispersed in Hank’s balanced salt solution (HBSS, pH = 7.3) and coated onto a flexible ITO before applying the PANI-G nanoelectrodes to perform an ES (Figure 1a). Due to hydrophobic nature of the ITO film, the PANI-G dispersion could not spread well on the ITO surface (Figure S1a). However, after treatment with oxygen plasma, the PANI-G dispersion was observed to spread evenly over the film (Figure S1b). The PANI-G nanoelectrode was ready for ES after its solvent was removed.

An atomic force microscopy (AFM) identified the morphology of the as-prepared PANI-G using a tapping mode. As shown in Figure 1b, the resulting PANI-G had a “classic” nanoflake-like structure with an average thickness of 1.7 nm, different from the AFM image of the ball milled graphite without addition of aniline (Figure S2). This confirmed the successful exfoliation of nanoscale graphene derivatives. This was also confirmed using a Raman spectroscopy (Figure S3). Four characteristic bands of polyaniline were visible at 1160 cm^−1^ (due to the C-C stretching of the benzenoid ring), 1330 cm^−1^ (because of the C=C stretching vibration in the quinonoid ring), 1490 cm^−1^ (attributed to the C=N stretching vibration), and 990 cm^−1^ (a characteristic peak associated with the vapor phase polymerized PANI via ball-milling), respectively. As soon as the PANI-G was confirmed, peaks at 1160 and 990 cm^−1^ were visible, while the bands at 1330 and 1490 cm^−1^ became overlapped with the characteristic D band and G band of graphene, respectively. The peak at 2700 cm^−1^ indicates the 2D band of graphene. Raman results confirmed the formation of PANI-G. A Fourier transform infrared spectroscopy (FTIR) of PANI-G (Figure S4) provided additional confirmation. The spectrum of PANI-G had some peaks that proved the presence of PANI. Two bands between 3300 cm^−1^ to 3500 cm^−1^ were associated with aN-H stretching vibration. Three peaks at 801, 1105, and 1239 cm^−1^ were assigned to the C-H vibration of benzene ring. Two bands at 1475 and 1573 cm^−1^ were attributed to a C=C vibration.

To improve the application of PANI-G in cell cultures and regeneration, we also tested the biocompatibility of PANI-G with PC12 cells. Figure 1c and Figure S5 show the CCK-8 result from PC12 cells incubated with different amounts of PANI-G. Relative cell viability did not change too much when the concentrations of PANI-G were less than 40 μg/mL. Cell viability was maintained at around 100%, even when the PANI-G concentration was 40 μg/mL and the cells were cultured over 72 h. This suggests the high biocompatibility of PANI-G at doses less than 40 μg/mL. Cell survival rate decreased to around 25% to 35% when 60 μg/mL PANI-G or more was added and the cells were incubated for 24 or 48 h. However, viability higher than 80% was seen when the culture time was set at 72 h, confirming again the low cytotoxicity of PANI-G, even at high doses. This increased cell viability with culture time may be attributed to the gradual adaptation of cells to the added materials.

The electrochemical properties of PANI-G were tested using acyclic voltammogram (CV) of PANI-G in HBSS (pH = 7.3). As a control, pristine graphite powder was treated under identical milling conditions, but without added polyaniline. The milled graphite and the pristine polyaniline were then coated to a glassy carbon (GC) electrode for electrochemical characterization. As shown in Figure 1d, an increase in capacitive currents was obtained at the CV of PANI-G, compared to that seen in the milled graphite and pure polyaniline (Figure S6). This indicates a significantly improvement in electroactivity and electrochemical capacitance of the PANI-G reported here.

### 2.2. Enhanced Axon Length of PC12 Cells after ES

In vitro electrical stimulation was applied to PC12 cells attached PANI-G nanoelectrodes as described in the experimental part. As shown in Figure S7, electrical stimulation was controlled using a constant pulsed potential (±500 mV/cm). As a negative control, PC12 cells were seeded on the ITO film, without the addition of ES.

**Figure 2:**
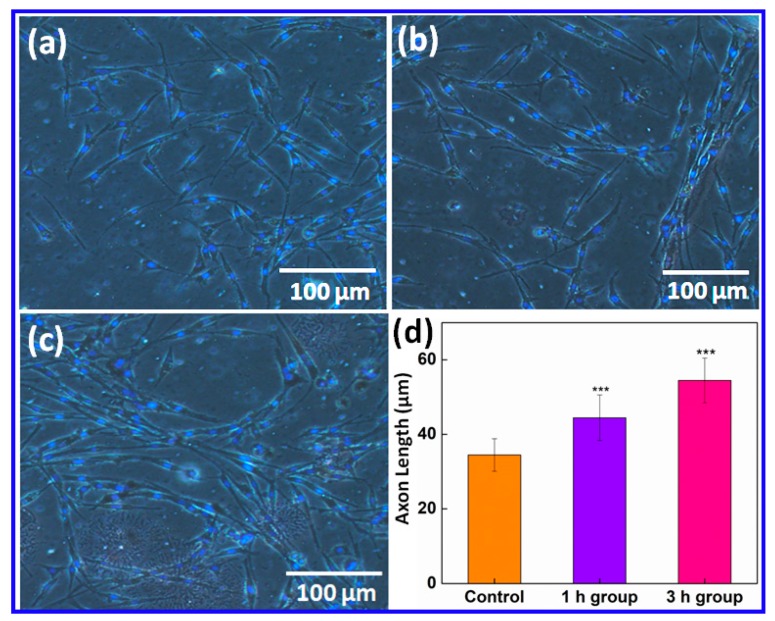
Microscopy images of (**a**) the control; (**b**) the PC12 cells after electrical stimulation for 1 h per day, over 5 days; (**c**) the PC12 cells after electrical stimulation for 3 h per day, over 5 days; and (**d**) changes to the average axon length of PC-12 cells after electrical stimulation (*** *p* < 0.001).

Cell axon length was measured as the distance between the edge of the nucleus and the end of the axon (Figure S8). As we can see from Figure 2, the average axon length of PC12 cells was significantly longer after ES treatment. Compared to the control tested (Figure 2a), the average axon length increased from 34 µm (in the control) to 44 µm after ES was applied for 1 h per day, over 5 days (*p* < 0.01, Figure 2b). A 30% increase in the axon length was observed (Figure 2d). When the ES condition was changed to 3 h per day, repeated over 5 days, the average axon length became further elongated to 54 µm (*p* < 0.005, Figure 2c). A dramatic 60% increase in the axon length was seen after ES treatment (Figure 2d). These results suggest the axon length of PC12 cells were improved significantly using ES and an appropriate nanoelectrode.

To better determine ES effects on cell axon growth, stimulation time was tested from 3 days to 7 days. As seen in Figure S9, the axon length of PC12 cells increased from 33 to 44 µm after stimulation time was increased from 3 days to 5 days. However, the axon length decreased to 35 µm after stimulation for 7 days. This suggests that 5 days ES is optimal.

### 2.3. Cell Density Changes of PC12 Cells after ES

**Figure 3:**
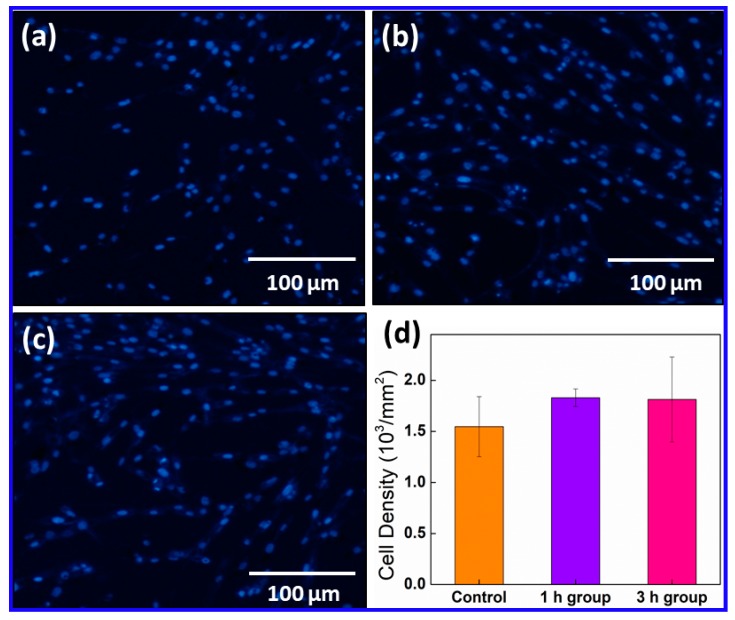
Immunofluorescence micrograph of DAPI-stained PC12 cells. (**a**) The control; (**b**) the PC12 cells after electrical stimulation for 1 h per day, repeated 5 days; (**c**) the PC12 cells after electrical stimulation for 3 h per day, repeated 5 days; and (**d**) changes in the cell density of PC-12 cells after electrical stimulation (*p* > 0.05).

Further tests on the impact of ES on the cell density of PC12 cells were performed. PC12 cell nuclei were stained with DAPI (blue, Figure 3). Fluorescent images of these stained cells were used to count the cell density (Figure S10). As shown in Figure 3d, the density of PC12 cells increased from 1.55×10^3^/mm^2^ in the control to 1.83 × 10^3^/mm^2^ after ES treatment for 1 h per day, over 5 days. However, the *p* value of the difference between the control and the ES group was higher than 0.05, indicating the difference was not significant. When ES frequency was increased to 3 h per day, the cell density was measured at 1.81 × 10^3^/mm^2^. Again, the *p* value was higher than 0.05. This suggests that the use of ES has no adverse impact on cell activity.

### 2.4. Improved Cell Wound Regeneration Ability of PC12 Cells after ES

**Figure 4:**
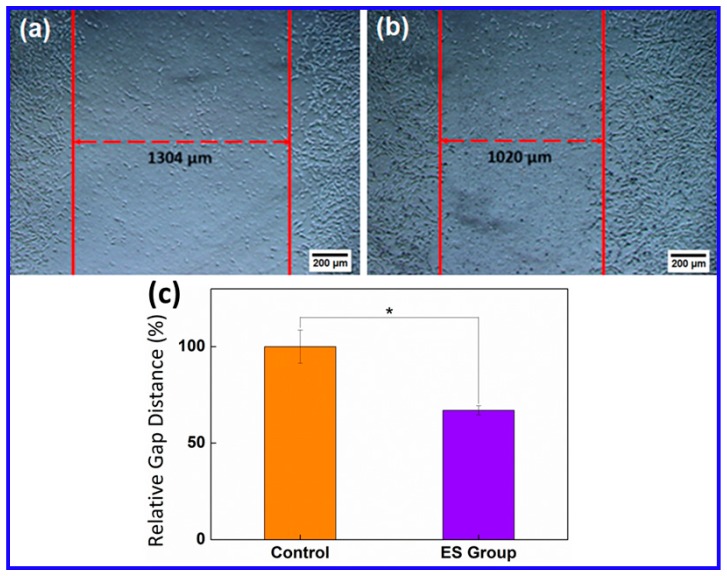
Microscopy images of the wound healing assay of (**a**) the control and (**b**) the PC12 cells after electrical stimulation. (**c**) Relative gap distance comparison between the control and the electrical stimulation group (* *p*< 0.05).

The impact of ES on the wound healing ability of PC12 cells was also tested by using a mechanically induced damage model. In a typical test, PC12 cell seeded PANI-G nanoelectrodes were mechanically scratched across their middle part (as shown in Figure 4a). The relative gap distance was found to be 1304 µm in the control test after 5 days. It was found that the gap distance significantly decreased to 1020 µm after 5 days of ES (Figure 4b). A decrease by 20% in the relative gap distance was achieved by ES (Figure 4c), suggesting the enhanced wound healing ability of PC12 cells activated by ES.

## 3. Discussion

Improvements to conventional ES techniques are highly needed since there is a demand for better nerve damage therapies [18]. As described in our previous reports and other reports elsewhere, the lack of high-electroactive stimulation electrodes is a key obstacle for the use of ES in clinics. In this work, we synthesized nitrogen edge-functionalized graphene (PANI-G) using aniline and graphite via an efficient polymerization-enhanced EFBM method. The PANI-G made with this technique exhibited high electroactivity and biocompatibility, important for the effective application of ES. To prepare scaffold nanoelectrodes for the ES on PC12 cells, PANI-G was cast onto flexible ITO films. A 60% increase in axon length and an increase in the number of regenerated PC12 cells that were damaged were observed.

During the application of ES, the extracellular region is driven to a more negative potential while the intracellular compartment is driven to a more positive potential. A charge is thus transferred across the cell membranes due to passive cell membrane properties and active ion channels. The Q_inj_ of the stimulation electrode is a limiting factor for the ES efficiency. The PANI-G reported here had a high electrochemical capacitance and electroactivity, which are attractive for the efficient application of ES. Particularly, the nitrogen edge-functionalized graphene preparation method reported here was an eco-friendly, facile, and efficient technique. Furthermore, good biocompatibility (more than 80% cell viability) of the PANI-G is also important for the ES application.

The ES process parameters used impacted the stimulation effects. Modifiable parameters include the applied potential waveform type, stimulation frequency, stimulation duration, and intensity of the applied electric field. (1) For example, the use of monophasic pulsingstimulation can be effective, but it causes bio-damage during long-term stimulation due to the great negative overpotentialscaused during pulsing. Compared to monophasic pulsing, biphasic pulsing creates lower negative overpotentials. During biphasic pulsing, the first stimulating phase elicits the initiation of an action potential, while the secondreversal phase reverses the direction of electrochemical processes occurring from the first phase. (2) It was observed that 1 h per day of ES was effective in promoting both motor and sensory axon regeneration [18]. Stimulation effects, however, were not dependent onits frequency. For an instance, a longer stimulation frequency had no impact on accelerating the outgrowth of sensory nerve [19]. In this study, we found that the cell density of PC12 cells remained unchanged, but the average axon length increased an extra 30%, when ES frequency was increased from 1 to 3 h per day. This work may provide how various stimulation goals can be meet, especially those focused on the axon length of neurons. (3) This work illustrated that stimulation duration played a greater role in nerve regeneration than previously thought. A significant improvement in the axon length of PC12 cells was obtained when the ES time was increased from 3 to 5 days. The axon length decreased when the ES time was shortened from 5 to 7 days. This may be attributed to the apoptosis of PC12 cells. Generally, most PC12 cells went through apoptosis and necrosis after culturing for 7 days [20]. In this work, however, PC12 cells maintained healthy cell morphology with significantly enhanced axon length, when compared to the control. This suggests that the ES treatment may improve the anti-aging ability of PC12 cells. (4) The intensity of the applied electrical field was also observed to influence the outcomes of stimulation. This work described that ±500 mV/cm was the best step-potential-range for the stimulated improvement of axon length of PC12 cells on a PANI-G nanoelectrode. Reports elsewhere suggest that 500 mV/cm can significantly enhance the neurite outgrowth of PC12 cells on conductive MEH-PPV: PCL electrospun nanofibers [21]. The neuronal differentiation of PC12 cells was reported elsewhere to be enhanced in an electric field intensity of 30 to 80 mV/mm [22]. Thus, the intensity of the applied electric field varied depending upon the types of both the ES electrodes used and the cells tested.

The function of the nervous system is dependent on the highly specific connections between neurons, achieved by neurite extension to correct targets [23]. The regeneration of axons is thus improved through functional recovery after nerve injury [24]. The changes to cells were mediated by the autocrine or paracrine action of neurotrophins, released by neurons during ES [25]. In this work, ES using PANI-G nanoelectrodes could repair a mechanically damaged PC12 cell model. This suggests that the ES technique described here also enhanced the autocrine or paracrine action of neurotrophins.

## 4. Materials and Methods

### 4.1. Synthesis and Characterization of PANI-G

The PANI-G was prepared via a polymerization-enhanced edge-functional ball-milling method. In a typical preparation, 10 mg graphite powders (Qingdao Haida Corporation, Qingdao, China) were placed in 10 µL aniline (Sigma-Aldrich, St. Louis, CA, USA). The mixture was milled in a planetary ball-milling machine (Nanjing NanDa Instrument Plant, Nanjing, China) set at a spin rate of 400 to 500 rpm for 6 to 8 h. The formed material was then transferred to a centrifuge tube containing 50 mL dehydrated alcohol. It was then centrifuged at 1000 rpm for 10 min to remove any remaining graphite and large sized-particles left in the precipitate. The supernatant was collected and re-centrifuged at 6000 rpm for 10 min to remove the remaining monomer left in the supernatant. The as-processed precipitate was then placed in a dialysis bag (molecular weight cutoff: 8000–14,000, Ye’yuan Biological Ltd., Shanghai, China). It was soaked in dehydrated alcohol for 72 h to further remove any remaining impurities. The final PANI-G materials were dispersed in HBSS (pH = 7.3, Gibco, Waltham, MA, USA) before testing it on biological samples.

The physiochemical properties of the as-prepared PANI-G nanosheets were characterized using Raman spectroscopy (Nicolet 6700, Thermo Scientific, Waltham, MA, USA) and atomic force microscopy (Digital Instruments Mutimode 8, Bruker, Billerica, MA, USA). The electrochemical properties and ES were performed using a CHI 760D electrochemical work station. Cyclic voltammetry of nanomaterials was carried out on a PANI-G coated glassy carbon (GC) electrode by dropping PANI-G onto a GC electrode (3 mm i.d.) and allowing it to be dried in air.

### 4.2. Fabrication of the PANI-G Nanoelectrodes

Indium tin oxide (ITO) coated conducting film (Zhuhai Kaiwei Technology Ltd., Zhuhai, China) was used as the supporting substrate for the PANI-G membrane. The ITO film was pre-treated by applying a radio-frequency glow discharge plasma [26] in an oxygen atmosphere for 10 s to clean its surface and improve its hydrophilicity. A total of 40 µg/mL PANI-G dispersion in HBSS was then spin-coated onto the ITO film. The resulting film remained in a 37 °C incubator overnight to remove the solvent. The amount of PANI-G in the as-prepared PANI-G/ITO electrode was approximately 20 µg/cm^2^.

### 4.3. Cell Culture

PC12 cells (Wuhan Procell Life Technology, Wuhan, China) were cultured in RPMI 1640 Medium (Gibco, Waltham, USA) supplemented with 10% heat-inactivated qualified fetal bovine serum (FBS, Gibco) and 0.2% gentamicin solution (50 mg/mL, Gibco). The cells were then incubated at 37 °C with 5% CO_2_ in a humid atmosphere. The PANI-G/ITO membrane was cut into circular pieces to fit into the culture wells. The membrane was sterilized by a 75% ethanol solution and exposed to ultraviolet light for 1 h, prior to its use in cell culturing. The biocompatibility of PANI-G was tested using a Cell Counting Kit-8 (CCK-8, Dojindo, Kumamoto, Japan) assay. This assay measures cell viability in cell proliferation and cytotoxicity using a sensitive colorimetric reaction. Dehydrogenase activities within cells reduce a highly water-soluble tetrazolium salt, WST-8 (developed by Dojindo). This forms a yellow-colored formazan dye soluble in the culture media. The amount of this dissolved dye can be used to count the number of living cells, which is proportional to the amount of dissolved dye. In this work, PC12 cells were seeded in a 96-well plate at a density of 5000 cells/well. Cells were pre-incubated for 24 h in a humidified incubator (at 37 °C, 5% CO_2_). PANI-G at various concentrations was added to the cell wells and co-cultured with nanomaterials over 24 to 72 h, before washing the cells with HBSS. A total of 10 µL CCK-8 solution was added into each well. The culture plate was then placed in the incubator over 0.5 to 2 h. The culture media absorbance was measured at 450 nm with a microplate reader (SpectraMax M5, Molecular Devices, San Jose, CA, USA).

### 4.4. Electrical Stimulation

PC12 cells were seeded on the PANI-G/ITO electrode to a density of 0.25 × 10^3^/mm^2^ and pre-cultured for 24 h. A platinum (Pt) electrode (ϕ = 0.5 mm) was contacted with the PANI-G/ITO electrode, while another Pt electrode was placed in the cell culture medium. The distance between these electrodes remained at 1 cm. An electrical field was applied to these cells using a double-pulsed potential chronoamperometry. A schematic set-up of the electrical stimulation is shown in Figure S11.

A forward 500 mV/cm potential and a reverse −500 mV/cm potential were applied. ES was carried out for 1 or 3 h each day. The stimulation was run for 3, 5, and 7 days, respectively. After this treatment, the medium was refreshed immediately to avoid its contamination. PC12 cells were then maintained in the incubator at 37 °C under 5% CO_2_. The properties of PC12 cells were measured after ES. As a negative control, PC12 cells were also cultured on pristine ITO film (that did not have a PANI-G coating) before testing with ES. Negative controls were also tested. PC12 cells were cultured in plates with no electrical stimulation.

### 4.5. Cell Morphology and Nucleus Staining

PC12 cells attached to PANI-G membranes were fixed by applying 4% paraformaldehyde (Aladdin, Shanghai, China) for 10 min and then permeabilized with 0.1% Triton-X 100 solution (Beyotime, Shanghai China) for 5 min. A total of 1 µg/mL 4’,6-diamidino-2-phenylindole (DAPI) was used to visualize nuclei. Superfluous dye stuff was washed away using PBS. A drop of antifade medium (Invitrogen, Waltham, MA, USA) was added to the cells, which was sealed with transparent nail polish. Images of samples were captured using fluorescent microscopy (Olympus, Shinjuku, Japan). The axon length of the PC12 cells was measured along the linear distance between the brink of a nucleus and the tip of an axon. The mean axon length was calculated by averaging the length of 300 measurements in each sample. These averages were then statistically analyzed across different samples. The mean cell density was calculated by imaging 3 fields in each sample and statistically analyzed. The cell density was countered using DAPI-stained fluorescence images that were analyzed with an Image-J software.

### 4.6. Statistical Analysis

A one-way analysis of variance (ANOVA) and post-hoc test statistically compared the changes in the axon length and cell density after electrical stimulation (Figure 2 and Figure 3). A *t*-test for each variable tested was calculated by comparing the wound healing ability of the ES group to a negative control. Statistical differences were calculated using a SPSS 16.0 software (Version 23, Armonk, New York, NY, USA). A * *p* < 0.05 was predetermined as significant difference in these calculations. A ** *p* < 0.01 indicates a higher significance and a *** *p* < 0.005 represents a very high significance.

## 5. Conclusions

This work reported a facile and efficient ES system, based on the PANI-G nanoelectrodes, to regenerate PC12 nerve cells. A high electroactive and biocompatible PANI-G nanomaterial was prepared via a polymerization-enhanced EFBM method. ES studies on PC12 cells attached to the PANI-G nanoelectrodes showed that ES can play a role in promoting PC12 cells regeneration. A 60% increase in axon length, with no adverse impact on the cell density and enhanced wound regeneration ability of PC12 cells after ES, was observed. The novel stimulation nanoelectrodes reported here provide a new choice for ES stimulation, facilitating the clinical application of ES in various fields, such as neuro traumatology.

## Supplementary Materials

Supplementary materials can be found at https://www.mdpi.com/1422-0067/20/8/2013/s1.

## Abbreviations

PANI-G	Polyaniline functionalized graphene
ES	Electrical stimulation
ITO	Indium tin oxide
HBSS	Hank’s balanced salt solution
AFM	Atomic force microscopy
FTIR	Fourier transform infrared spectroscopy
CV	Cyclic voltammogram
Pt	Platinum
DAPI	4’,6-diamidino-2-phenylindole
