# Supplementary material for: Polyaniline Functionalized Graphene Nanoelectrodes for the Regeneration of PC12 Cells via Electrical Stimulation
Source: Int J Mol Sci. 2019 Apr 24;20(8):2013. doi: 10.3390/ijms20082013 (PMC6515035; doi:10.3390/ijms20082013)
Supplement: Supplementary file 1 [file ijms-20-02013-s001.pdf]

Supplemental material

Polyaniline functionalized graphene nanoelectrodes for the regeneration of PC12 cells via electrical stimulation

Zheng Zheng [1], Libin Huang [1], Lu Yan [1], Feng Yuan [1], Lefeng Wang [1], Ke Wang [1], Tom Lawson [2], Mimi Lin [1,*] and Yong Liu[1,*]

[1] Laboratory of Nanoscale Biosensing and Bioimaging, School of Ophthalmology and Optometry, School of Biomedical Engineering, Wenzhou Medical University, 270 Xueyuanxi Road, Wenzhou, Zhejiang 325027, China

[2] ARC Center of Excellence for Nanoscale BioPhotonics, Macquarie University, Sydney, NSW 2109, Australia

[*] Emails: linmimi815@wmu.edu.cn (M.L.); yongliu@wmu.edu.cn （Y.L.）

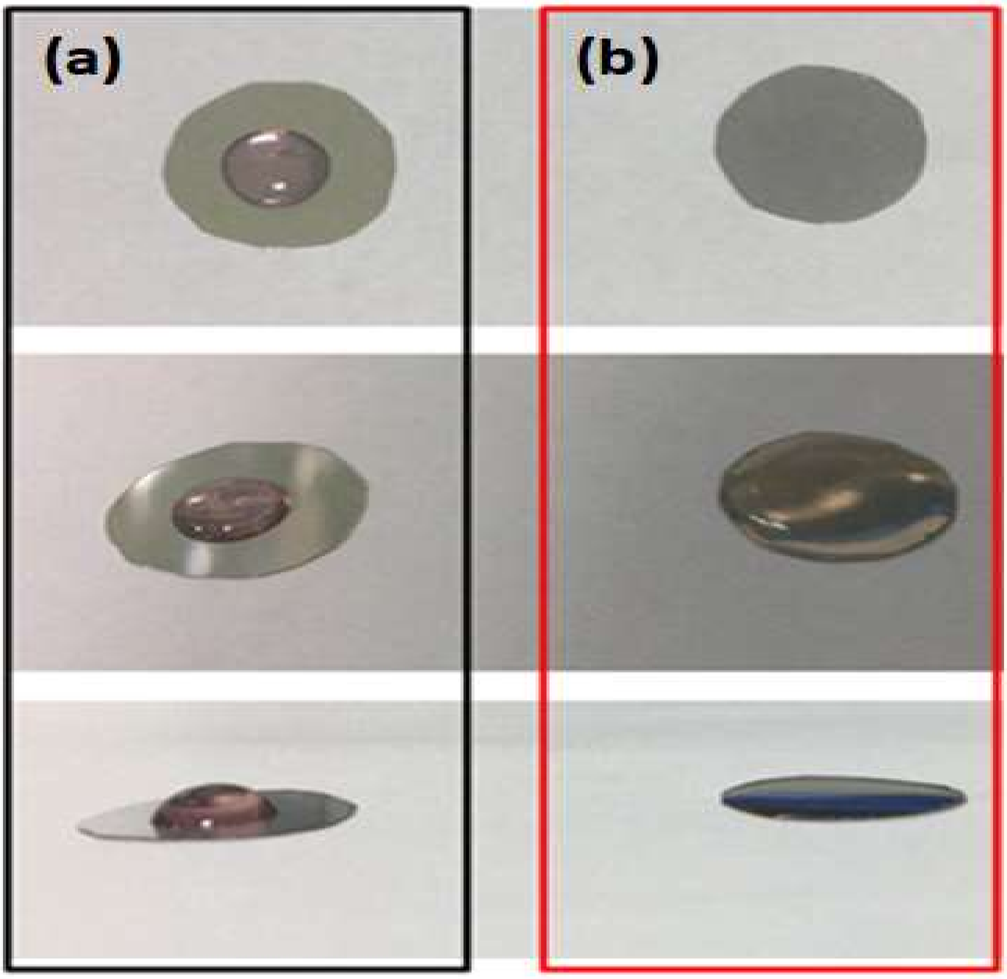

Figure S1. Digital photos of the PANI-G/HBSS dispersion on (**a**) an untreated ITO film, (**b**) an ITO film treated by oxygen plasma.

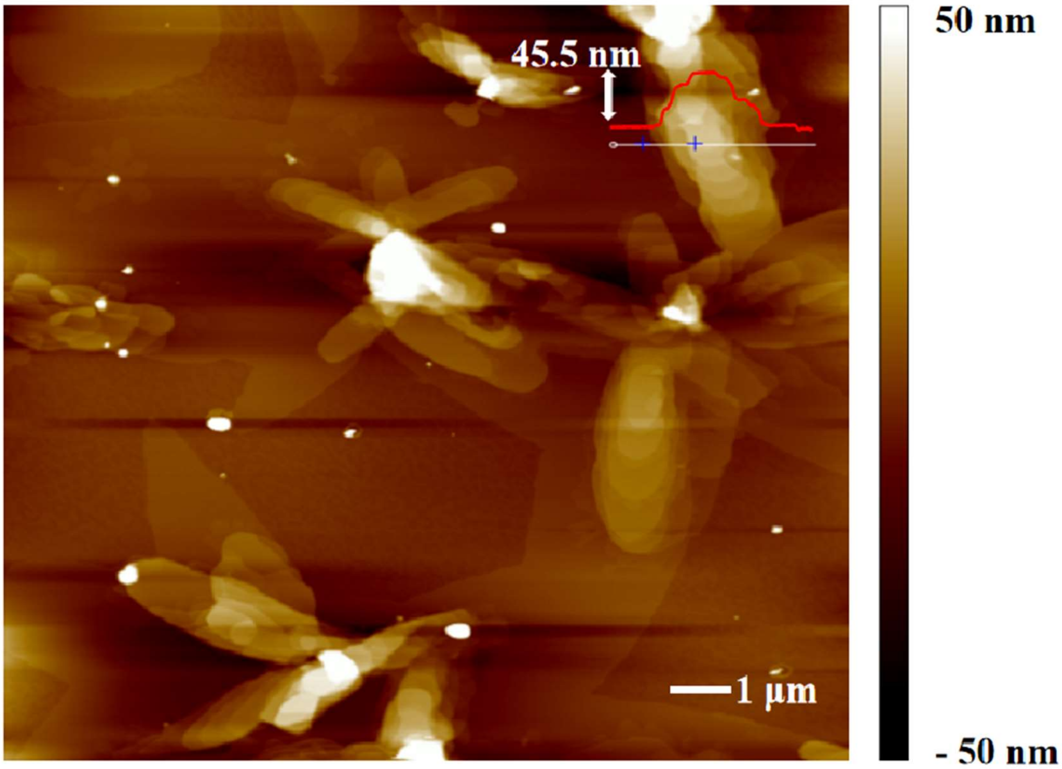

Figure S2. AFM micrograph of the pristine graphite after ball-milling without the addition of aniline under the identical conditions.

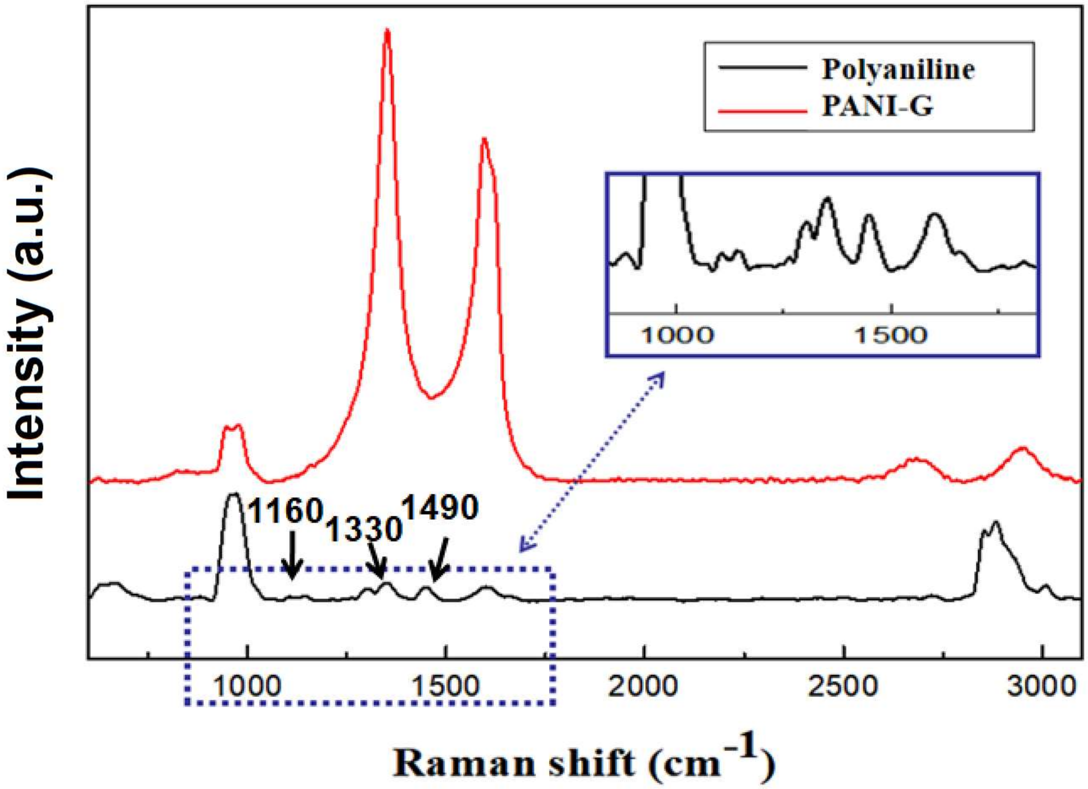

Figure S3. Raman spectra of polyaniline functionalized graphene (PANI-G), compared to that of polyaniline.

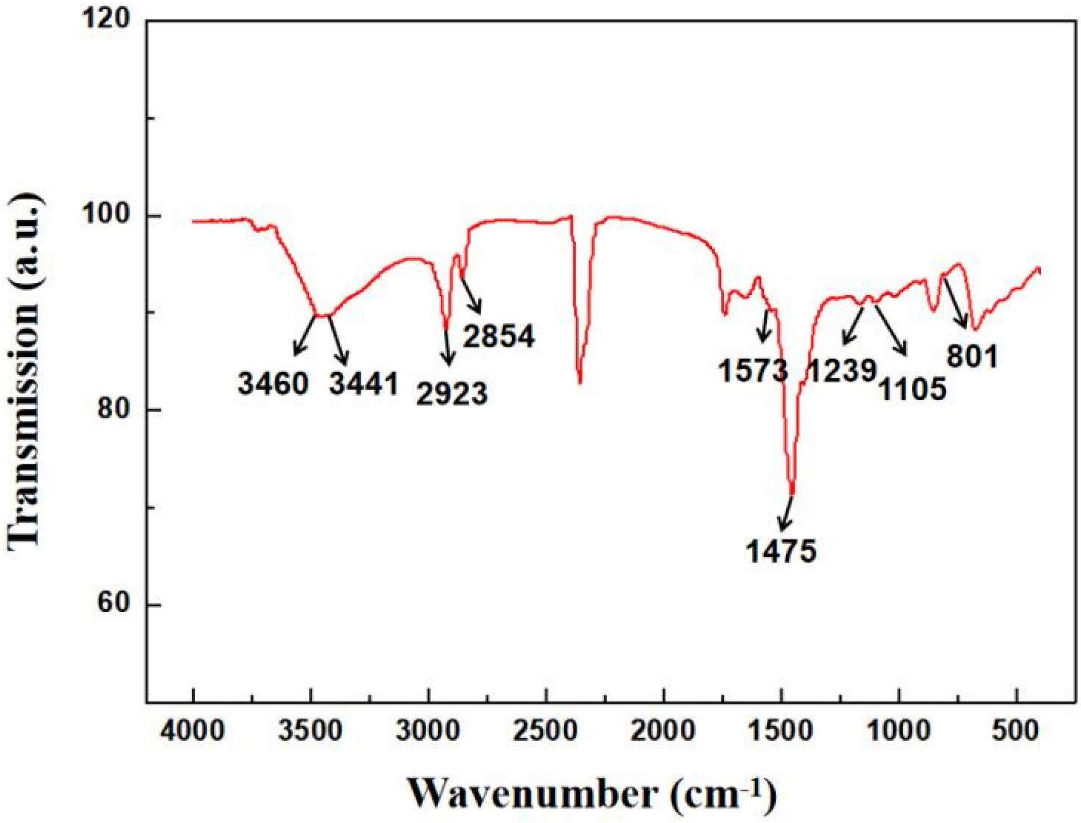

Figure S4. FTIR spectrum of polyaniline functionalized graphene (PANI-G).

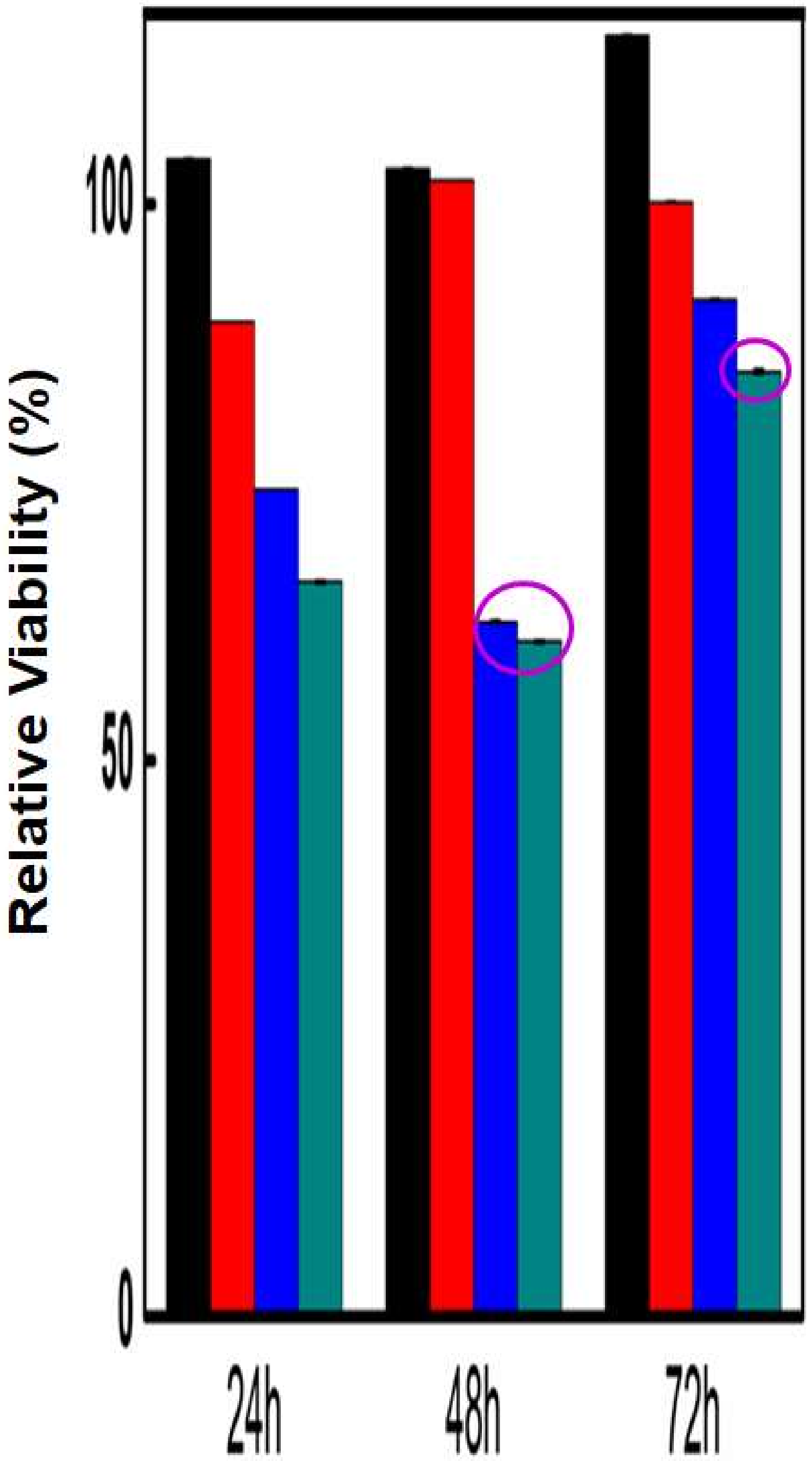

Figure S5. Magnified image of Figure 1c. Error bars are too small to see since the difference between various groups was very small. Purple circles indicate some visible error bars.

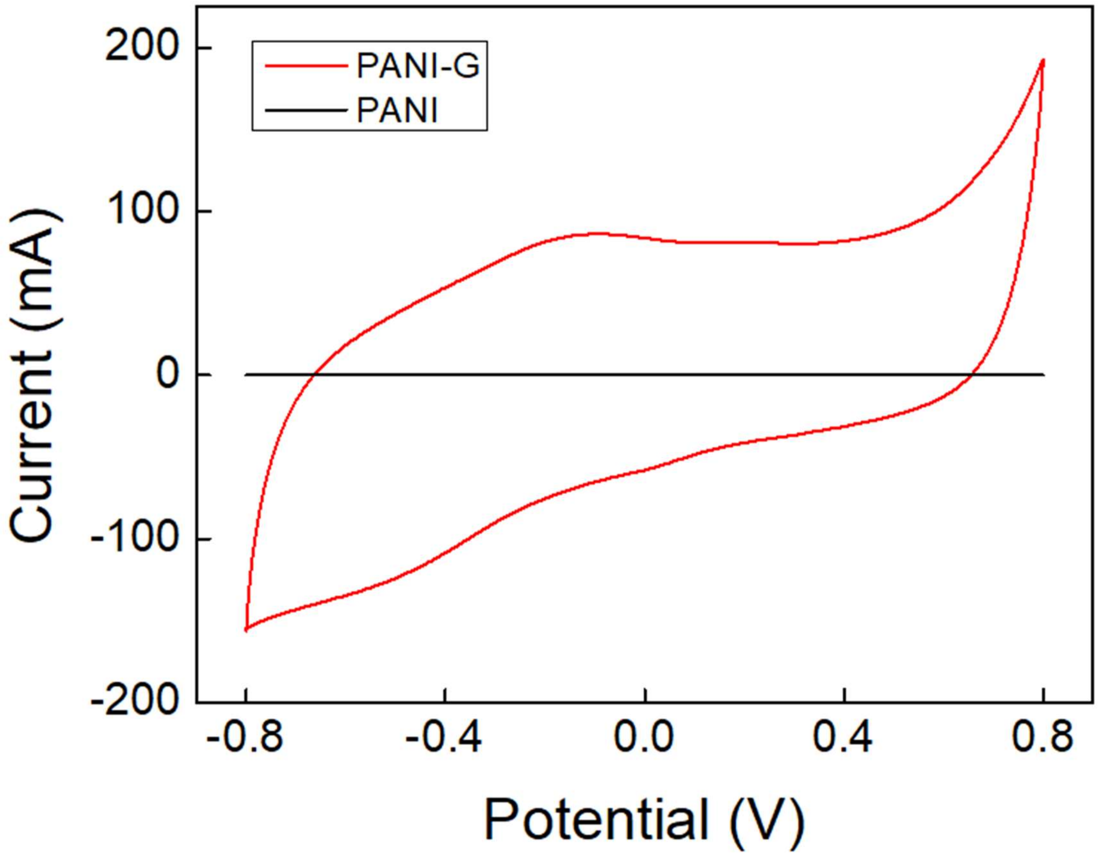

Figure S6. Cyclic voltammograms of the PANI-G coated on the glassy carbon electrode, compared to the PANI prepared from aniline via ball milling.

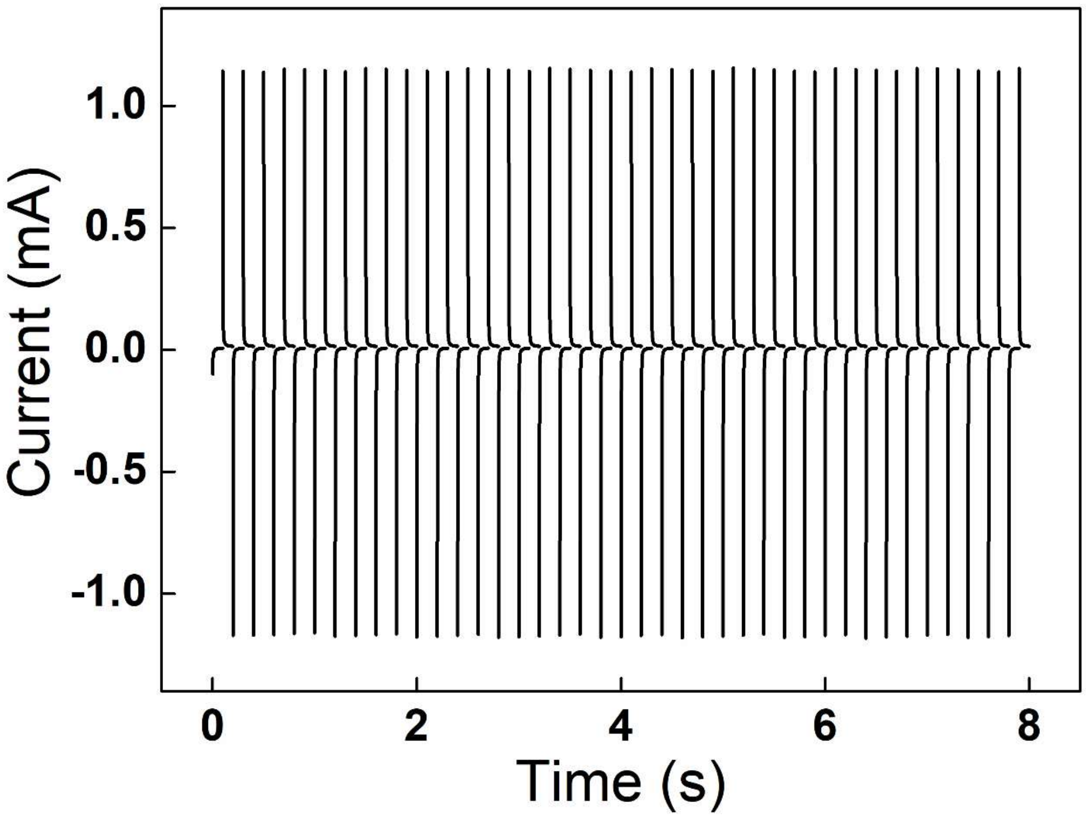

Figure S7. Current−time response during electrical stimulation on the PAG nanoelectrode, controlled by a double-potential step chronoamperometry. The applied potential steps were +500 and −500 mV/cm.

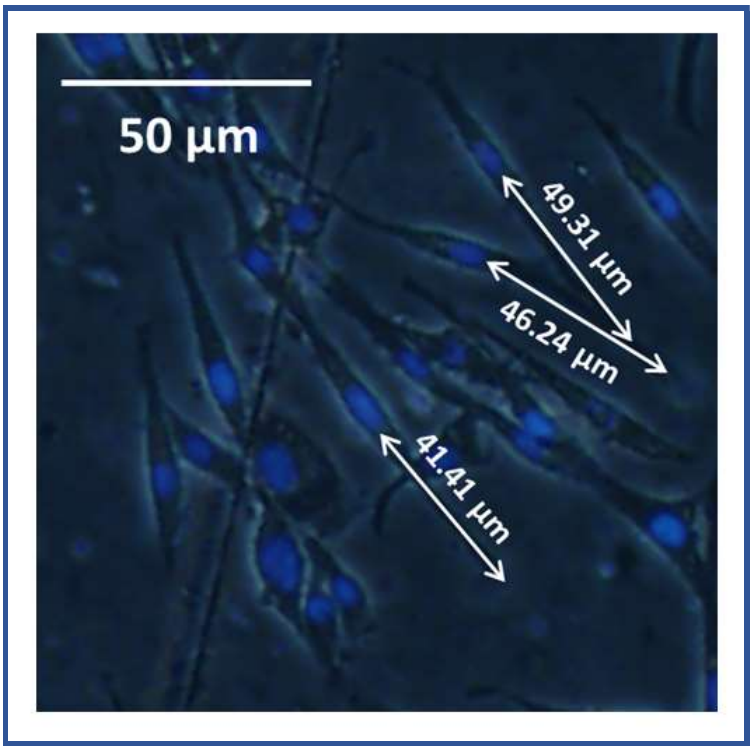

Figure S8. Fluorescent micrograph illustrating the measurement on the axon length of PC12 cells.

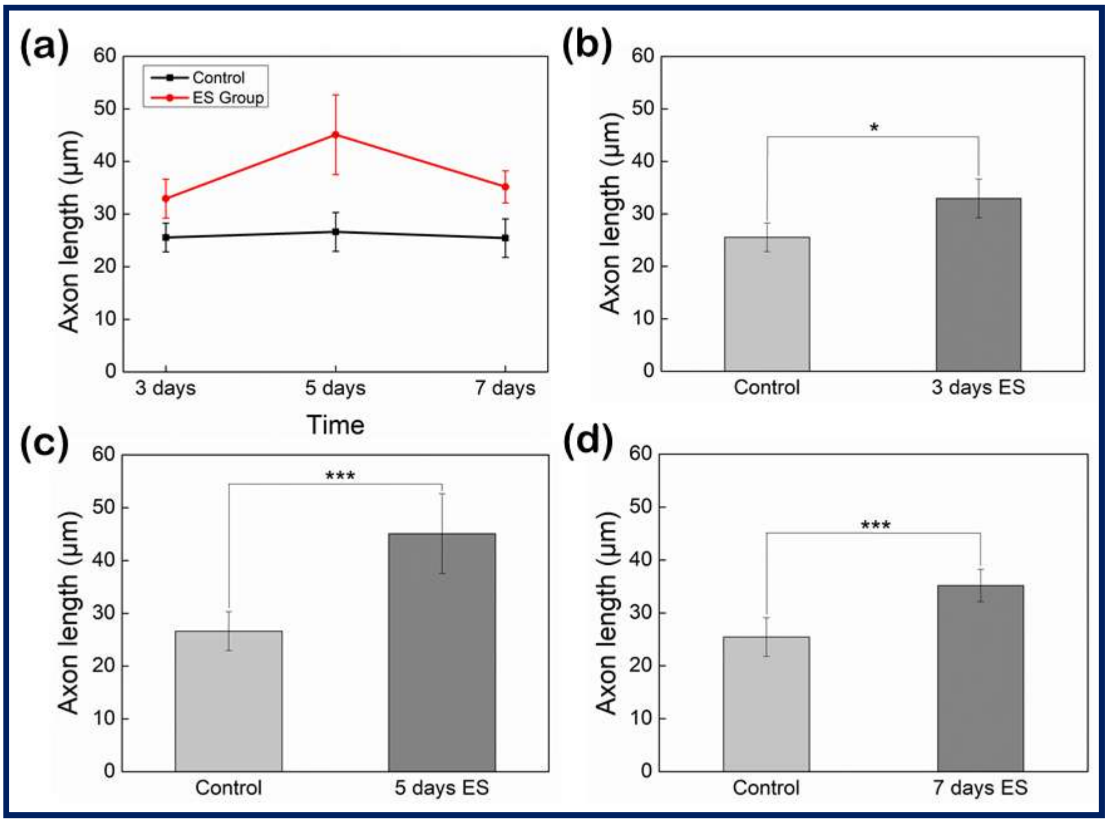

Figure S9. Axon length changes of PC12 cells after electrical stimulation for 1 h per day, repeated for 3, 5, and 7 days, respectively (*$p < 0.05$, ***$p < 0.001$).

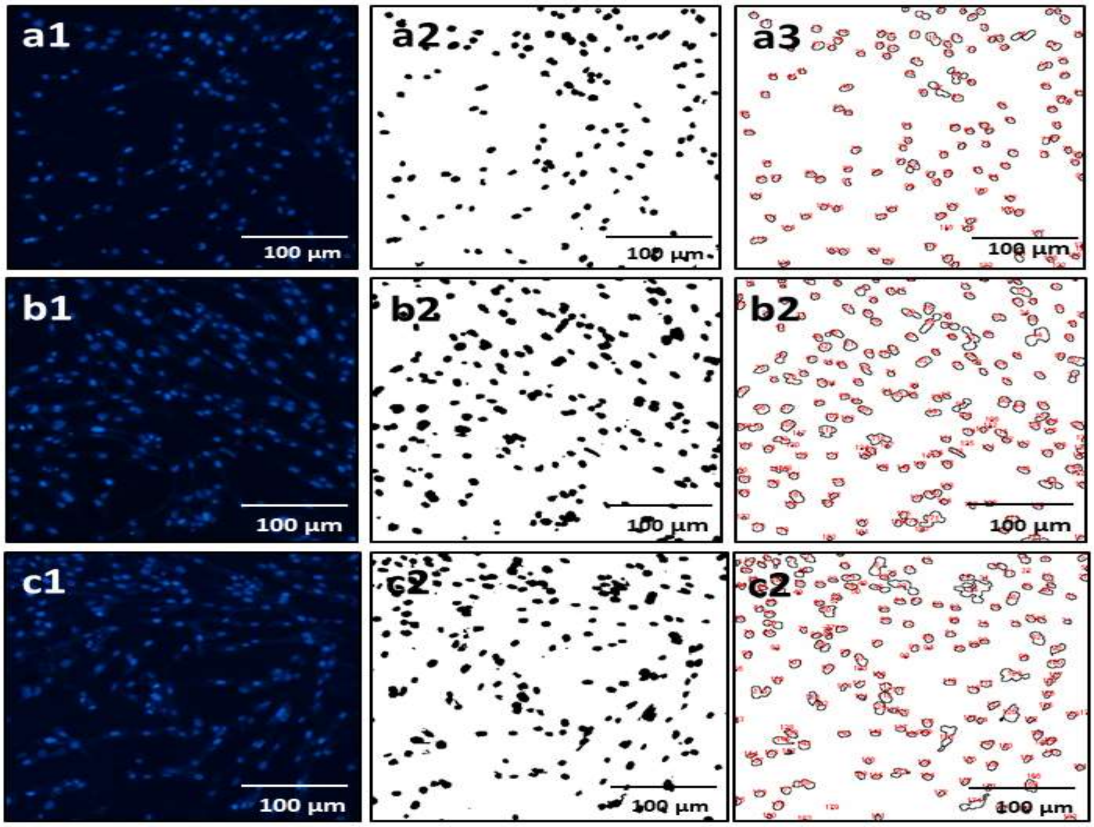

Figure S10. Immunofluorescence images of DAPI-stained PC-12 cells after electrical stimulation. (**a1**) is the pristine fluorescence image; (**a2**) is the image conversion of (**a1**); (**a3**) is the image accounting on how to calculate the number of PC-12 cells on the PAG nanoelectrode; (**a1**–**a3**) are the corresponding images of the control; (**b1**–**b3**) are the images of PC 12 cells after electrical stimulation for 1 h per day, repeated for 5 days; and (**c1**–**c3**) are the images of PC 12 cells after electrical stimulation for 3 h per day, repeated for 5 days.

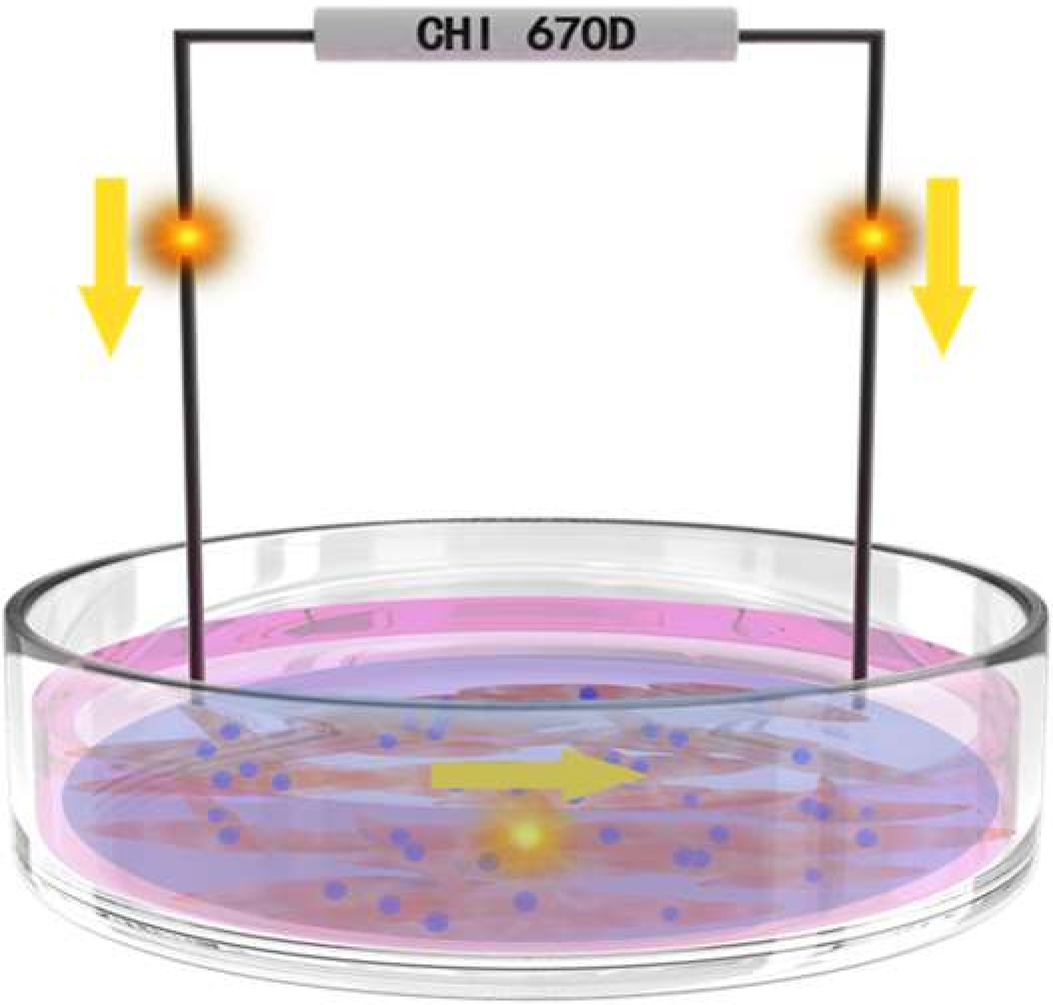

Figure S11. Schematic set-up of the electrical stimulation system.
